# FGFR-1 amplification in metastatic lymph-nodal and haematogenous lobular breast carcinoma

**DOI:** 10.1186/1756-9966-31-103

**Published:** 2012-12-27

**Authors:** Eleonora Brunello, Matteo Brunelli, Giuseppe Bogina, Anna Caliò, Erminia Manfrin, Alessia Nottegar, Marco Vergine, Annamaria Molino, Emilio Bria, Francesco Massari, Giampaolo Tortora, Sara Cingarlini, Serena Pedron, Marco Chilosi, Giuseppe Zamboni, Keith Miller, Guido Martignoni, Franco Bonetti

**Affiliations:** 1 Department of Pathology and Diagnostic, University of Verona, P.le Ludovico Scuro n. 10, Verona, 37134, Italy; 2 Ospedale Sacro Cuore, Negrar, Verona, Italy; 3 King’s College Hospital NHS Foundation Trust, London, United Kingdom; 4 Medical Oncology dO, Azienda Ospedaliera Universitaria Integrata, Verona, Italy; 5 Medical Oncology dU, Azienda Ospedaliera Universitaria Integrata, Verona, Italy; 6 United Kingdom National External Quality Assessment Service (UKNeqas), London, United Kingdom

**Keywords:** Lobular breast carcinoma, Metastases, FGFR-1 amplification, In situ hybridization

## Abstract

**Background:**

Lobular breast carcinoma usually shows poor responsiveness to chemotherapies and often lacks targeted therapies. Since FGFR1 expression has been shown to play pivotal roles in primary breast cancer tumorigenesis, we sought to analyze the status of FGFR1 gene in a metastatic setting of lobular breast carcinoma, since promising FGFR1 inhibitors has been recently developed.

**Methods:**

Fifteen tissue metastases from lobular breast carcinomas with matched primary infiltrative lobular breast carcinoma were recruited. Eleven cases showed loco-regional lymph-nodal and four haematogenous metastases.

FGFR-1 gene (8p12) amplification was evaluated by chromogenic in situ hybridization (CISH) analysis. Her-2/neu and topoisomerase-II**α** gene status was assessed. E-cadherin and Hercept Test were also performed. We distinguished amplification (>6 or cluster of signals) versus gains (3–6 signals) of the locus specific FGFR-1 gene.

**Results:**

Three (20%) primary lobular breast carcinomas showed >6 or cluster of FGFR1 signals (amplification), six cases (40%) had a mean of three (range 3–6) chromogenic signals (gains) whereas in 6 (40%) was not observed any abnormality. Three of 15 metastasis (20%) were amplified, 2/15 (13,4%) did not. The ten remaining cases (66,6%) showed three chromogenic signals.

The three cases with FGFR-1 amplification matched with those primary breast carcinomas showing FGFR-1 amplification. The six cases showing FGFR-1 gains in the primary tumour again showed FGFR-1 gains in the metastases. Four cases showed gains of FGFR-1 gene signals in the metastases and not in the primary tumours. Her-2/neu gene amplification was not observed in all cases but one (6%) case. Topoisomerase-IIα was not amplified in all cases.

**Conclusions:**

1) a subset of metastatic lobular breast carcinoma harbors FGFR-1 gene amplification or gains of chromogenic signals; 2) a minor heterogeneity has been observed after matching primary and metastatic carcinomas; 3) in the era of tailored therapies, patients affected by the lobular subtype of breast carcinoma with FGFR1 amplification could be approached to the new target biological therapy such as emerging FGFR-1 inhibitors.

## Introduction

In breast carcinoma, the response to chemotherapy or targeted therapies varies according to histology [1]. Although effective regimens are currently established for invasive ductal carcinoma, the treatment efficacy and the prognosis of other minor types of breast cancer are not adequately developed. The lobular histotype, the second most common subtype of breast carcinomas (15%), actually show poor responsiveness to available chemotherapies, thus rarely implying tailored therapies for patients treatments [2,3]. Defining the relationship between each histological type and the clinicopathological response to therapies is essential to optimizing individualized treatment. Overall, classical lobular breast carcinoma is orphan of good standard medical therapies with recognizable high level of efficacy at any clinical end-points such as overall survival, disease free-survival or progression free-survival [1,4]. In fact, the Her-2/neu gene is rarely amplified in lobular carcinoma, avoiding trastuzumab therapeutic chances for most the patients, and even worse, the topoisomerase-IIa is constantly not-amplified [2], thus predicting high chances of chemo-resistance to anthracyclines. In this poor context of medical therapies, new promising predictive biomarkers, giving chances in selecting appropriate patients suitable for receiving new effective regimens, are needed [5,6].

Among several biomarker studied by different technical approaches, Reis-Filho et al. studied a small series of primary lobular breast carcinomas and reported six cases to be with gains of the locus specific FGFR-1 gene, thus suggesting that receptor FGFR-1 inhibitors may be useful as therapeutics [7]. Data on the efficacy of anti-FGFR-1 inhibitor do seem promising [8-10]. The study reported herein was designed to analyze the status of FGFR-1 gene in a consecutive series of lobular breast carcinoma with primary and matched lymph-nodal and haematogenous metastases from lobular breast carcinomas, given no data are currently available on the FGFR-1 gene status in a metastatic setting of lobular breast carcinomas.

The importance to assess new biomarker in a metastatic setting is of note because clinical trials are usually designed with patients affected by an advanced/metastatic disease.

## Material and methods

### Tissue samples

Fifteen tissue metastases from lobular breast carcinomas with matched primary infiltrative lobular breast carcinoma where recruited from the file of the Department of Pathology and Diagnostic, University of Verona and Hospital SacroCuore, Negrar, Verona, Italy. Eleven cases showed loco-regional lymph-nodal and four haematogenous metastases.

We used tissue samples from human participants. All tissue blocks have been previously declaired to be available for the purposes of the actual study by the Istitutional Review Board (study conducted according to the principles expressed in the Declaration of Helsinki). Our institutional review board and the ethics committee approved the original human work that produced the tissue samples. All processing in obtaining the material has been performed after a written informed consent. Full name Ethic/Institutional Review Board: Nucleo Ricerca&Innovazione, University of Verona.

Formalin-fixed and paraffin-embedded tumor blocks were retrieved from archivial file. Whole tissue sections were cut from each block at 5 μm thickness and were stained with haematoxylin and eosin. From these sections one representative of the whole tumor was evaluated. All cases were reviewed: only tumor with complete lack of ductal structure and with typical lobular features have been admitted to the study.

### Immunohistochemical analysis

Estrogen (ER rabbit, SP1, 1:50, Neomarkers) and progesterone (PgR 636, 1:150, Dako) receptors were evaluated. Ki67% (MM1, 1:50, Novocastra) were also assessed. Ki67% was considered low when scored <20%, medium >20 x <50% and high when >50% of neoplastic nuclei. E-cadherin and GATA-3 immunostaining were available for each tumor.

According to the recommendations from the manufacturer of the HercepTest kit (DAKO, Glostrup, Denmark), tissue sections mounted on slides and stored at room temperature were stained within 4 to 6 weeks from sectioning to maintain antigenicity.

HER-2 immunoexpression was assessed by using Hercept test (Dako) according to datasheet recommandations.

### Fluorescence in situ hybridization (FISH) analysis

We performed FISH analysis on whole tissue section for Her-2/neu and topoisomerase-IIα amplification.

FISH was performed by using the PathVysion Her-2/neu (Abbott/Vysis Inc, Rome) and topoisomerase-IIα (Dako, Milan) DNA probe kits from. The technical procedure has been previously described [11]. Two hundred neoplastic nuclei were counted.

Specimens were determined to be amplified according to FDA and ASCO/CAP cut-offs values. Polysomy for chromosome 17 was defined when the ratio was around 1 in tumors having 3 or more locus-specific HER-2/*neu* and centromeric 17 signals, similarly for topoisomerase-IIα gene status.

The slides were examined using an Olympus BX61 (Olympus, Milan) with appropriate filters. The signals were recorded with a CCD camera (Olympus). Slides were also digitalized by D-Sight/Fluo (Menarini/VisiaImaging, Florence).

### Chromogenic in situ hybridization analysis (CISH)

FGFR1 gene (8p12) amplification was evaluated by chromogenic in situ hybridization (CISH) (ZytoLight, Bremerhaven, Germany) analyses.

CISH was performed in all cases applying the protocol of the CISH technology of ZytoVysion. This technique allows advanced specificity and less background due to the unique ZytoVision Repeat Subtraction Technique and is characterized by high sensitivity due to enzyme-coupled polymers for the detection of FGFR-1 gene gains. We followed steps of the datasheet ZytoDot-2C protocol.

In normal cells, two distinct dot-shaped signals per nucleus are observed (disomic pattern). We distincted among cases showing FGFR-1 gains two groups: amplification if the number of chromogenic signals was >6 per 60 neoplastic nuclei or showing cluster of signals versus simple gains when the mean score number of chromogenic signals set in between 3 and 5 per 60 neoplastic nuclei.

## Results

In situ results are summarized in Table 1.

**Table 1:** Metastatic lobular breast carcinoma with matched primary tumours: FGFR1 gene status by molecular analysis

**FGFR-1 gene status by chromogenic in situ hybridization (CISH)**					**FISH analysis**		**Immunophenotyping**			
					**Topoisomerase-IIα**	**Her-2/neu**				
**Primary breast carcinoma**			**Tissue metastases**		**Both primary and metastases**		**HER2**	**ER**	**PR**	**Ki67%**
**1**	infiltrative lobular breast carcinoma	**amplified**	lymph-nodal	**amplified**	not-amplified	not-amplified	0	positive	positive	high
**2**	infiltrative lobular breast carcinoma	**amplified**	lymph-nodal	**amplified**	not-amplified	not-amplified	0	positive	positive	low
**3**	infiltrative lobular breast carcinoma	**amplified**	haematogenous	**amplified**	not-amplified	amplified (in mts)	1+	positive	positive	low
**4**	infiltrative lobular breast carcinoma	gains	lymph-nodal	gains	not-amplified	not-amplified	0	positive	positive	low
**5**	infiltrative lobular breast carcinoma	gains	lymph-nodal	gains	not-amplified	not-amplified	0	positive	positive	medium
**6**	infiltrative lobular breast carcinoma	gains	lymph-nodal	gains	not-amplified	not-amplified	0	positive	positive	low
**7**	infiltrative lobular breast carcinoma	gains	lymph-nodal	gains	not-amplified	not-amplified	0	positive	positive	low
**8**	infiltrative lobular breast carcinoma	gains	lymph-nodal	gains	not-amplified	not-amplified	0	positive	positive	low
**9**	infiltrative lobular breast carcinoma	gains	lymph-nodal	gains	not-amplified	not-amplified	0	positive	positive	low
**10**	infiltrative lobular breast carcinoma	disomic	lymph-nodal	gains	not-amplified	not-amplified	0	positive	positive	low
**11**	infiltrative lobular breast carcinoma	disomic	lymph-nodal	gains	not-amplified	not-amplified	0	positive	positive	low
**12**	infiltrative lobular breast carcinoma	disomic	lymph-nodal	gains	not-amplified	not-amplified	0	positive	positive	low
**13**	infiltrative lobular breast carcinoma	disomic	haematogenous	gains	not-amplified	not-amplified	0	positive	positive	low
**14**	infiltrative lobular breast carcinoma	disomic	haematogenous	disomic	not-amplified	not-amplified	0	positive	positive	low
**15**	infiltrative lobular breast carcinoma	disomic	haematogenous	disomic	not-amplified	not-amplified	0	positive	positive	low

Amplified: >6 or cluster of signals; gains: 3–5 signals.

*ER*: estrogen receptor, *PR*: progesteron receptor.

### Morpho-Immunophenotypical analysis

**Figure 1:**
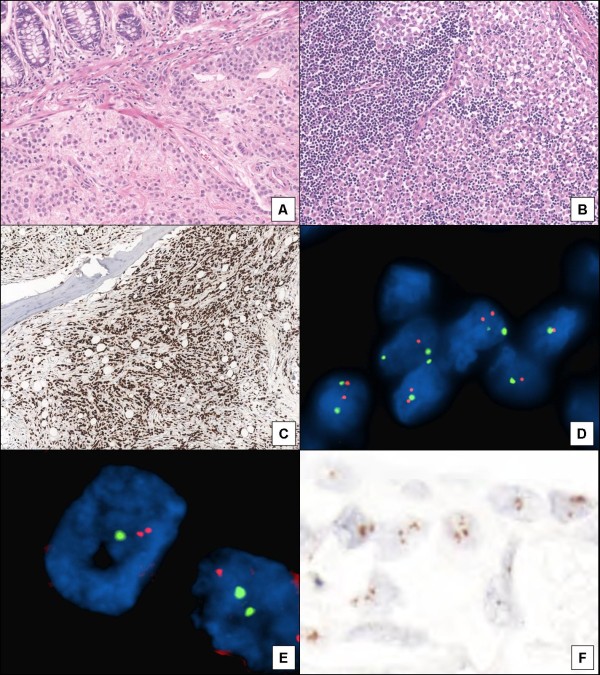
Metastatic lobular breast carcinoma to the colon (A, H&E), to the lymph-node (B, H&E) and to the bone (C, GATA-3 immunoexpression); FISH analysis showing absence of Her-2/neu (D) and topoisomerase-IIα (E) gene amplification, avoiding selection of the patients to targeted and individualized chemiotherapies. FGFR-1 gene amplification in a metastasis of lobular breast carcinoma by CISH analysis (F).

Morphological appearance and E-cadherin negative staining confirmed all tumors to be pure lobular infiltrative carcinoma. Thirteen cases out of 15 (86,6%) were defined as classical, 1 solid (6,6%) and 1 as pleomorphic lobular carcinoma (6,6%). Four and eleven cases were respectively characterized by haematogenous (1 ovarian, 1 colonic, 1 cerebral and 1 from bone) (Figure 1A, C) and lymph-nodal (Figure 1B) metastases. 

All cases showed positive estrogen and progesterone immunoexpression (primary tumours and metastases). Ki67% was low for all cases except for the pleomorphic type and one classic subtype, which showed respectively an high and a medium proliferative rate index. Hercept test scored 0 in 13 cases and 1+ in two cases. GATA-3 was always positive.

### Fluorescence in situ hybridization (FISH) analysis

Her-2/neu gene amplification was not observed in all cases (Figure 1D) but one (6,6%) case in a metastasis. Topoisomerase-IIα was not amplified in all cases (Figure 1E).

### Chromogenic in situ hybridization (CISH) analysis

Three (20%) primary lobular breast carcinomas showed >6 or cluster of FGFR-1 signals (amplification) (Figure 1F), six cases (40%) had a mean of three (range 3–6) chromogenic signals (gains) whereas in 6 (40%) was not observed any abnormality. Three of 15 metastasis (20%) were amplified, 2/15 (13,4%) did not. The ten remaining cases (66,6%) showed three chromogenic signals.

The three cases with FGFR-1 amplification matched with those primary breast carcinomas showing FGFR-1 amplification. The six cases showing FGFR-1 gains in the primary tumour again showed FGFR-1 gains in the metastases. Four cases showed gains of FGFR-1 gene signals in the metastases and not in the primary tumours.

## Discussion

The data reported herein, show that: 1) FGFR-1 amplification is observed in a subset of lymph-nodal and haematogenous metastases from lobular breast carcinoma; 2) minor heterogeneity is scored in matched primary and metastatic lobular breast carcinomas; 3) in the era of tailored therapies, patients affected by the lobular subtype of breast carcinoma with FGFR-1 amplification may be considered a potential patients’ subset benefiting from FGFR-1 inhibitor.

The efficacy use of endocrine therapies for hormone receptor-positive breast cancer and trastuzumab and lapatinib for targeting HER2-positive tumors has placed the way for the clinical development of other metastatic breast cancer targeted therapies [12]. Conversely, the benefit of anti-VEGF (vascular endothelial growth factor) monoclonal antibody in the metastatic setting, is still under investigation, as well as new HER2-targeted agents and VEGF-targeted agents, dual epidermal growth factor receptor/HER2-targeted agents, multitargeted tyrosine kinase inhibitors, and mammalian target of rapamycin and poly (ADP-ribose) polymerase 1 inhibitors [12]. These anticancer agents are being tested in clinical trials with the potential of addressing unmet therapeutic needs in the metastatic patient population [13].

In the breast cancer scenario, Massabeau et al. evaluated the role of FGFR1 and its ligand, the fibroblast growth factor 2 in determining the response to chemoradiotherapy [14]. Among the low/intermediate grade tumors, FGFR-1 negative tumors did not respond to chemoradiotherapy, compared with tumors expressing FGFR-1 among which, almost one half had a good response. Among the low and intermediate grade breast cancers, the FGFR-1 negative tumors were resistant to chemoradiotherapy. They concluded that the expression of FGFR-1 in patients’ biopsies may serve as a marker of response to chemoradiotherapy. Turner et al. concluded that amplification and overexpression of FGFR1 may be a major contributor to poor prognosis in luminal-type breast cancers, driving anchorage-independent proliferation and endocrine therapy resistance [15]. In our study we found a subset of lobular breast carcinoma, be characterized by FGFR-1 amplification or gains of chromogenic signals, not only in primary tumours but also in the metastatic tissue. In this context, patients affected by lobular breast carcinomas and characterized by gains/amplification of FGFR-1 molecule, could receive effective regimens (predictive biomarker) with FGFR-1 inhibitors (targeted therapy). Differently, lobular breast carcinomas usually show absence of Her-2/neu and topoisomerase-IIα gene amplification (according to both FDA or ASCO/CAP cut-offs [11]), thus patients constantly lack the ad hoc predictive rationale for receiving common chemotherapy that includes anthracyclines; in addition, invasive lobular carcinomas commonly underexpress Her-2 showing lack of tailored scheme of therapies.

The efficacy of anti-FGFR-1 inhibitor is increasing also in carcinomas arising from other organs. Interestingly, Dutt et al. found gains of FGFR-1 gene in a subset of lung adenocarcinomas and squamous lung carcinomas and notably they demonstrated that a non-small cell lung carcinoma cell line harbouring focal amplification of FGFR-1 is dependent on FGFR-1 activity for cell growth, as treatment of this cell line either with FGFR1-specific shRNAs or with FGFR small molecule enzymatic inhibitors did lead to cell growth inhibition [16]. They concluded that FGFR-1 may represent a promising therapeutic target in non-small cell lung cancer and even better in the orphan subtype of lung carcinoma such as the squamous.

Intratumoral heterogeneity can lead to underestimation of the tumor genomics portrayed from single tumoral samples and may present challenges to personalized-medicine and biomarker development. Intratumor heterogeneity may foster tumor adaptation and therapeutic failure [17]. We found no significant heterogeneity in matched primary and metastatic lobular breast carcinomas in regard to FGFR-1 gains or amplification. The predictive biomarker may be assessed on metastatic tissue or in primary carcinomas, and the predictiveness to anti-FGFR-1 inhibitor is prone to be similar.

The assessment of the FGFR-1 gene status may be performed on formalin-fixed and paraffin embedded materials, actually by using commercially available kit. The design of new clinical trials have to take in account these clustered molecular patterns in order to make an appropriate correlation between abnormalities of the FGFR-1 gene and predictiveness of emerging drug efficacy. The clinical significance in between amplification (>6 chromogenic signals) versus simple gains (3–6 signals) may be assessed differently; we actually do not know if anti-FGFR1 inhibitors work equally. Polyploidy of nuclei due to disruption of the mitotic machinery may be the reasons of simple gains of cromogenic signals, differently to true gene amplification where additional gains of signals are more than reference probes (true gene amplification). We clustered these two molecular groups similarly to those distinct in the Her-2/neu assessment when overall gene copy number is scored.

The FGFR-1 overexpression is already been noted, however no data is available on its presence in a metastatic setting. Reis-Filho et al. studied eighteen infiltrative lobular breast carcinomas and reported gains of FGFR-1 by arrayCGH in five cases and validated specific gains of genomic material after in situ hybridization analysis [7]. Courjal et al. studied by Southern blotting a total of 1875 breast tumor DNAs with 26 probes mapping at 15 distinct chromosomal localizations [18]. They identified a group of carcinomas with amplifications at 11q13 and/or 8p12 and was predominantly composed of estrogen receptor-positive tumors and presented a large proportion of lobular cancers. Coamplifications of the 11q13 and 8p12 regions are common in breast carcinomas, suggesting synergy between the amplicons [19,20]. Gelsi-Boyer et al. found genomic “turbulence” at 8p11 in a subset of lobular breast carcinomas [21] whereas Adelaide et al. described a recurrent chromosome translocation breakpoint near the 8p12 locus [22]. Jacquemier et al. observed that overexpression of FGFR-1 to be associated with small, well-differentiated diploid breast cancers [23]. Elbauomy Elsheikh et al. suggested that FGFR-1 amplification may be an independent predictor of overall survival in patients affected by breast carcinoma [24].

The fibroblast growth factor (FGF) signaling axis is increasingly implicated in tumorigenesis [25] and chemoresistance. Several small molecule FGF-receptor (FGFR) kinase inhibitors are currently in clinical development [5,8,26], however, the predominant activity of the most advanced of these agents is against the kinase insert domain receptor, which compromises the FGFR selectivity [27,28]. Most of studies did not encounter the lobular subtypes of breast carcinoma when evaluating FGFR-1 gene status. Shiang et al. suggested that FGFR-1 amplification or protein overexpression in breast cancers may be an indicator for brivanib treatment, where it may have direct anti-proliferative effects in addition to its’ anti-angiogenic effects [29]. Gru et al. found a twofold increase in FGFR1 amplification in invasive breast carcinoma versus pure ductal carcinoma in situ, and they observed a significant reduction of the disease-free survival in amplified versus unamplified invasive breast carcinoma [30]. Balko et al. suggested that the addition of FGFR inhibitors to ER-targeted therapy will yield a superior antitumor effect [31]. Jang et al. reported the increased frequency of FGFR1 amplification in invasive carcinomas compared with pure in situ ductal carcinoma [32]. They suggested a role for FGFR1 amplification in the progression of breast cancer including in situ-to-invasive transition. Only 3.2% of their cases had lobular features, thus we can not compare our findings. Massabeau et al. observed a correlation in between patients showing response to chemotherapy and the FGFR-1 positive findings by immunophenotypical analysis at cancerous tissue level [14]. Moelens et al. reported around 20-30% of invasive ductal breast carcinoma harboring FGFR-1 amplification (ratio >1.3) [33]. Again, no lobular have been analyses.

Overall, emerging interest is present at any level of translational research in regard to FGFR-1 as a biomarker predictive of responsiveness to targeted and/or personalized therapies. In the era of tailored therapies, patients affected by the lobular subtype of breast carcinoma with FGFR-1 amplification could be approached to the new target biological therapy such as FGFR-1 inhibitor, with promising clinical efficacy. The lobular infiltrative breast carcinoma may become an ex orphan cancer of targeted therapy. In our study, we observed the presence of FGFR-1 genomic abnormalities such as gains and amplification in a significant subset of metastatic lobular breast carcinoma, with clear implications for targeted therapy use.

## Competing interests

The authors declare that they have no competing interests.

## Authors’ contribution

EB drafted the manuscript, MB interpreted the molecular analyses and drafted the manuscript, GB set up the database, AC interpreted the molecular analysis, EM participated in the sequence alignment, AN recruited tissue samples, MC recruited tissue samples, AM reviewed the criticisms, EB recruited the clinical information, FM recruited the clinical information, GT recruited the clinical information, SC recruited the clinical information, SP performed the technical experiments, MC interpreted the immunophenotypical analysis, GZ recruited tissue samples, KM verified the distribution of HER2 analysis, GM participated in the sequence alignment, FB approved, followed and managed all processing steps of research. All the authors read and approved the final manuscript.

## References

[B1] Berruti A Generali D Kaufmann M Puztai L Curigliano G Aglietta M Gianni L Miller WR Untch M Sotiriou C International expert consensus on primary systemic therapy in the management of early breast cancer: highlights of the fourth symposium on primary systemic therapy in the management of operable breast cancer, Cremona, Italy (2010) J Natl Cancer Inst Monogr 2011 2011 147 151 10.1093/jncimonographs/lgr037 22043063

[B2] Brunello E Brunelli M Manfrin E Nottegar A Bersani S Vergine M Molino A Fiorio E Chilosi M Gobbo S Martignoni G Bonetti F Classical lobular breast carcinoma consistently lacks topoisomerase-IIalpha gene amplification: implications for the tailored use of anthracycline-based chemotherapies Histopathology 2012 60 482 488 10.1111/j.1365-2559.2011.04067.x 22168383

[B3] Vergine M Brunelli M Martignoni G Brunello E Miller K Pecori S Bersani S Chilosi M Menestrina F Manfrin E Bonetti F Suitability of infiltrative lobular breast carcinoma for anti-human epidermal growth factor receptor 2 treatment after the ASCO/CAP and 2009 St Gallen International Expert Consensus meeting Histopathology 2010 57 935 940 21166708 10.1111/j.1365-2559.2010.03716.x

[B4] Cristofanilli M Gonzalez-Angulo A Sneige N Kau SW Broglio K Theriault RL Valero V Buzdar AU Kuerer H Buccholz TA Hortobagyi GN Invasive lobular carcinoma classic type: response to primary chemotherapy and survival outcomes J Clin Oncol 2005 23 41 48 15625359 10.1200/JCO.2005.03.111

[B5] Gozgit JM Wong MJ Moran L Wardwell S Mohemmad QK Narasimhan NI Shakespeare WC Wang F Clackson T Rivera VM Ponatinib (AP24534), a multitargeted pan-FGFR inhibitor with activity in multiple FGFR-amplified or mutated cancer models Mol Cancer Ther 2012 11 690 699 10.1158/1535-7163.MCT-11-0450 22238366

[B6] Patel RR Sengupta S Kim HR Klein-Szanto AJ Pyle JR Zhu F Li T Ross EA Oseni S Fargnoli J Jordan VC Experimental treatment of oestrogen receptor (ER) positive breast cancer with tamoxifen and brivanib alaninate, a VEGFR-2/FGFR-1 kinase inhibitor: a potential clinical application of angiogenesis inhibitors Eur J Cancer 2010 46 1537 1553 10.1016/j.ejca.2010.02.018 20303261 PMC2927957

[B7] Reis-Filho JS Simpson PT Turner NC Lambros MB Jones C Mackay A Grigoriadis A Sarrio D Savage K Dexter T FGFR1 emerges as a potential therapeutic target for lobular breast carcinomas Clin Cancer Res 2006 12 6652 6662 10.1158/1078-0432.CCR-06-1164 17121884

[B8] Ayers M Fargnoli J Lewin A Wu Q Platero JS Discovery and validation of biomarkers that respond to treatment with brivanib alaninate, a small-molecule VEGFR-2/FGFR-1 antagonist Cancer Res 2007 67 6899 906 10.1158/0008-5472.CAN-06-4555 17638901

[B9] Andre F Bachelot TD Campone M Dalenc F Perez-Garcia JM Hurvitz SA Turner NC Rugo HS Shi MM Zhang Y Kay A Yovine AJ Baselga J A multicenter, open-label phase II trial of dovitinib, an FGFR1 inhibitor, in FGFR1 amplified and non-amplified metastatic breast cancer J Clin Oncol 2011 508 Suppl 508

[B10] Koziczak M Holbro T Hynes NE Blocking of FGFR signaling inhibits breast cancer cell proliferation through downregulation of D-type cyclins Oncogene 2004 23 3501 3508 10.1038/sj.onc.1207331 15116089

[B11] Brunelli M Manfrin E Martignoni G Bersani S Remo A Reghellin D Chilosi M Bonetti F HER-2/neu assessment in breast cancer using the original FDA and new ASCO/CAP guideline recommendations: impact on selecting patients for herceptin therapy Am J Clin Pathol 2008 129 907 911 10.1309/MD79CDXN1D01E862 18480007

[B12] Perez EA Spano JP Current and emerging targeted therapies for metastatic breast cancer Cancer 2012 118 3014 25 10.1002/cncr.26356 22006669

[B13] Baselga J Novel agents in the era of targeted therapy: what have we learned and how has our practice changed? Ann Oncol 2008 19 Suppl 7 vii281 vii288 18790967 10.1093/annonc/mdn433

[B14] Massabeau C Sigal-Zafrani B Belin L Savignoni A Richardson M Kirova YM Cohen-Jonathan-Moyal E Mégnin-Chanet F Hall J Fourquet A The fibroblast growth factor receptor 1 (FGFR1), a marker of response to chemoradiotherapy in breast cancer? Breast Cancer Res Treat 2012 134 259 266 10.1007/s10549-012-2027-3 22438050

[B15] Turner N Pearson A Sharpe R Lambros M Geyer F Lopez-Garcia MA Natrajan R Marchio C Iorns E Mackay A FGFR1 amplification drives endocrine therapy resistance and is a therapeutic target in breast cancer Cancer Res 2010 70 2085 2094 10.1158/0008-5472.CAN-09-3746 20179196 PMC2832818

[B16] Dutt A Ramos AH Hammerman PS Mermel C Cho J Sharifnia T Chande A Tanaka KE Stransky N Greulich H Inhibitor-sensitive FGFR1 amplification in human non-small cell lung cancer PLoS One 2011 6 e20351 10.1371/journal.pone.0020351 21666749 PMC3110189

[B17] Gerlinger M Rowan AJ Horswell S Larkin J Endesfelder D Gronroos E Martinez P Matthews N Stewart A Tarpey P Intratumor heterogeneity and branched evolution revealed by multiregion sequencing N Engl J Med 2012 366 883 892 10.1056/NEJMoa1113205 22397650 PMC4878653

[B18] Courjal F Cuny M Simony-Lafontaine J Louason G Speiser P Zeillinger R Rodriguez C Theillet C Mapping of DNA amplifications at 15 chromosomal localizations in 1875 breast tumors: definition of phenotypic groups Cancer Res 1997 57 4360 4367 9331099

[B19] Kwek SS Roy R Zhou H Climent J Martinez-Climent JA Fridlyand J Albertson DG Co-amplified genes at 8p12 and 11q13 in breast tumors cooperate with two major pathways in oncogenesis Oncogene 2009 28 1892 1903 10.1038/onc.2009.34 19330026 PMC2722962

[B20] Karlsson E Waltersson MA Bostner J Perez-Tenorio G Olsson B Hallbeck AL Stal O High-resolution genomic analysis of the 11q13 amplicon in breast cancers identifies synergy with 8p12 amplification, involving the mTOR targets S6K2 and 4EBP1 Genes Chromosomes Cancer 2011 50 775 787 10.1002/gcc.20900 21748818

[B21] Gelsi-Boyer V Orsetti B Cervera N Finetti P Sircoulomb F Rouge C Lasorsa L Letessier A Ginestier C Monville F Comprehensive profiling of 8p11-12 amplification in breast cancer Mol Cancer Res 2005 3 655 667 10.1158/1541-7786.MCR-05-0128 16380503

[B22] Adelaide J Chaffanet M Mozziconacci MJ Popovici C Conte N Fernandez F Sobol H Jacquemier J Pebusque M Ron D Translocation and coamplification of loci from chromosome arms 8p and 11q in the MDA-MB-175 mammary carcinoma cell line Int J Oncol 2000 16 683 688 10717235 10.3892/ijo.16.4.683

[B23] Jacquemier J Adelaide J Parc P Penault-Llorca F Planche J deLapeyriere O Birnbaum D Expression of the FGFR1 gene in human breast-carcinoma cells Int J Cancer 1994 59 373 378 10.1002/ijc.2910590314 7927944

[B24] Elbauomy Elsheikh S Green AR Lambros MB Turner NC Grainge MJ Powe D Ellis IO Reis-Filho JS FGFR1 amplification in breast carcinomas: a chromogenic in situ hybridisation analysis Breast Cancer Res 2007 9 R23 10.1186/bcr1665 17397528 PMC1868920

[B25] Ugolini F Adelaide J Charafe-Jauffret E Nguyen C Jacquemier J Jordan B Birnbaum D Pebusque MJ Differential expression assay of chromosome arm 8p genes identifies Frizzled-related (FRP1/FRZB) and Fibroblast Growth Factor Receptor 1 (FGFR1) as candidate breast cancer genes Oncogene 1999 18 1903 1910 10.1038/sj.onc.1202739 10086345

[B26] Tenhagen M van Diest PJ Ivanova IA van der Wall E van der Groep P Fibroblast growth factor receptors in breast cancer: expression, downstream effects, and possible drug targets Endocr Relat Cancer 2012 19 R115 29 10.1530/ERC-12-0060 22508544

[B27] Xian W Pappas L Pandya D Selfors LM Derksen PW de Bruin M Gray NS Jonkers J Rosen JM Brugge JS Fibroblast growth factor receptor 1-transformed mammary epithelial cells are dependent on RSK activity for growth and survival Cancer Res 2009 69 2244 2251 10.1158/0008-5472.CAN-08-3398 19258500 PMC11888581

[B28] Gavine PR Mooney L Kilgour E Thomas AP Al-Kadhimi K Beck S Rooney C Coleman T Baker D Mellor MJ Brooks AN Klinowska T AZD4547: an orally bioavailable, potent, and selective inhibitor of the fibroblast growth factor receptor tyrosine kinase family Cancer Res 2012 72 2045 2056 10.1158/0008-5472.CAN-11-3034 22369928

[B29] Shiang CY Qi Y Wang B Lazar V Wang J Fraser Symmans W Hortobagyi GN Andre F Pusztai L Amplification of fibroblast growth factor receptor-1 in breast cancer and the effects of brivanib alaninate Breast Cancer Res Treat 2010 123 747 755 10.1007/s10549-009-0677-6 20024612

[B30] Gru AA Allred DC FGFR1 amplification and the progression of non-invasive to invasive breast cancer Breast Cancer Res 2012 14 116 10.1186/bcr3340 23151501 PMC4053127

[B31] Balko JM Mayer IA Sanders ME Miller TW Kuba MG Meszoely IM Wagle N Garraway LA Arteaga CL Discordant cellular response to presurgical letrozole in bilateral synchronous ER + breast cancers with a KRAS mutation or FGFR1 gene amplification Mol Cancer Ther 2012 11 2301 2305 10.1158/1535-7163.MCT-12-0511 22879364 PMC3682668

[B32] Jang MH Kim EJ Choi Y Lee HE Kim YJ Kim JH Kang E Kim SW Kim IA Park SY FGFR1 is amplified during the progression of in situ to invasive breast carcinoma Breast Cancer Res 2012 14 R115 10.1186/bcr3239 22863309 PMC3680930

[B33] Moelans CB de Wegers RA Monsuurs HN Maess AH van Diest PJ Molecular differences between ductal carcinoma in situ and adjacent invasive breast carcinoma: a multiplex ligation-dependent probe amplification study Cell Oncol (Dordr) 2011 34 475 482 21547576 10.1007/s13402-011-0043-7 PMC3219861

